# Editorial: Bacterial diseases in poultry: Biology, virulence and prevention in the age of reduced antibiotic use

**DOI:** 10.3389/fvets.2023.1189315

**Published:** 2023-03-31

**Authors:** Thi Thu Hao Van, Peter M. Smooker, Shubiao Wu, Zuowei Wu

**Affiliations:** ^1^School of Science, RMIT University, Bundoora, VIC, Australia; ^2^School of Environmental and Rural Science, University of New England, Armidale, NSW, Australia; ^3^Veterinary Microbiology and Preventive Medicine, Iowa State University, Ames, IA, United States

**Keywords:** bacterial disease, poultry, non-antibiotic alternatives, antibiotic resistance, campylobacter, *Salmonella*

Poultry plays an important role in global food security. In poultry production, antibiotics have been used to prevent and treat various bacterial diseases, but their use leads to residues in animal products and the development of antibiotic resistant bacteria. Therefore, non-antibiotic alternatives for the treatment of bacterial diseases are needed. This Research Topic focuses on the current knowledge of the bacteria responsible for poultry diseases and approaches to prevent those diseases in the post-antibiotic era.

Spotty Liver Disease is a significant disease in poultry as it causes significant losses and mortalities in layer hens (1). The causative agent of the disease was identified and named *Campylobacter hepaticus* in 2016 (2). Recently, *C. bilis* was also confirmed as a causative agent of SLD (3). Muralidharan, Quinteros et al. developed an enzyme-linked immunosorbent assay (ELISA) for screening antibody responses to *C. hepaticus* exposure in hens. A fragment of filamentous hemagglutinin (FHA1628-1899) was found to be immunogenic and specific to *C. hepaticus* and used for the assay. The assay specificity and sensitivity were 95 and 93%, respectively and it is useful for SLD epidemiological and vaccine development studies.

Muralidharan, Huang et al. used this ELISA assay to investigate the prevalence of *C. hepaticus* specific antibodies among commercial free-range layers in Australia. The PCR specific for the detection of *C. hepaticus* DNA was also used. Interestingly, four out of five farms without a history of SLD, *C. hepaticus* specific antibodies were detected with seroprevalence as high as 41% on one of the farms. In three SLD-infected farms, egg production reduction and mortalities were comparatively mild, concurrent with the low prevalence of *C. hepaticus* DNA and anti-*C. hepaticus* antibodies in flocks, suggesting the variability in virulence of *C. hepaticus* strains. The study demonstrates the usefulness of ELISA and PCR for SLD control.

Van et al. found that *C. hepaticus* infection impacted the microbiota of chickens. In trials, the *C. hepaticus* challenged group had lower bacterial diversity. Some SCFA-producing bacteria such as *Faecalibacterium, Bifidobacterium* and *Megamonas* were significantly reduced in the challenged groups compared to the unchallenged control group. Although SLD induction affected the gut microbiota of chickens, their small intestine morphology was not noticeably affected. Approaches to improve the birds' gut health during SLD outbreaks, such as through diet, SCFA addition, and keeping the causes of stress to a minimum, may help in the management of SLD.

With the emergence of antibiotic-resistant bacteria in animal production systems, finding alternatives to treat bacterial diseases is urgently required (4). Keerqin et al. isolated bacteriophages from poultry environments to target *Clostridium perfringens* in chickens. Necrotic enteritis (NE) is a significant bacterial disease in poultry, and *C. perfringens* strains that express NetB toxin (5) are causative agents. A cocktail containing three bacteriophages isolated from the study was capable of lysing four known disease-inducing *C. perfringens* strains *in vitro*. Broilers fed with the phage cocktail significantly reduced intestinal necrotic lesions when challenged with *C. perfringens* compared to the control group and the lesion scores between birds treated with the bacteriophages and the unchallenged birds were not statistically different. The isolated bacteriophages had 96.7% similarity to the closest characterized *Clostridium* bacteriophage. In another aspect of bacteriophage, Wang et al. studied the relationship between the newly discovered *Riemerella anatipestifer* 50K genomic island and the *R. anatipestifer* phage RAP44. *R. anatipestifer* mainly infects ducklings, geese, and turkeys. The phage RAP44 genome was integrated into *R. anatipestifer* chromosome and the 50K genomic island was integrated, excised, and cyclised automatically, showing evidence for the evolution of *R. anatipestifer* genomes.

Two major diseases caused by *Salmonella* spp. in poultry are pullorum disease (PD) and fowl typhoid (FT), with *S. pullorum* and *S. gallinarum* as their pathogens (6). To identify sustainable means to control these diseases, Xu et al. evaluated the potential of oregano essential oil (OEO) in the drinking water of birds to suppress disease. Birds were supplied with OEO either throughout the trial (prophylactic dose) or from the day after the challenge to the end of the trial (therapeutic dose). Birds treated with a prophylactic dose of OEO had similar weight to, while those given a therapeutic dose were lower than, the uninfected birds. Importantly, this was the case irrespective of the species. The administration of OEO either before or after the challenge significantly reduced colonization compared to untreated birds.

In a further study evaluating natural products against *Salmonella* species, Jiang et al. used an extract known as Dahuang Qinyu San (DQS), isolated from Chinese medicinal plants. A semi-biomimetic extract of the active ingredients, termed SEDQS was evaluated. It was demonstrated that SEDQS reduced growth rates of *S. enteriditis*, isolated from a goose farm, increased the permeability of the cell wall, and had an inhibitory effect on the activity of certain enzymes. Furthermore, the expression levels of five virulence genes were shown to be significantly reduced. It was concluded that SEDQS has the potential for use as an alternative to antibiotics for the control of *S. enteritidis*. In another study related to *S. enteritidis*, Wellawa et al. used bioluminescent *S. enteritidis* strains carrying the *lux* operon to follow the kinetics of colonization in chickens. After the challenge in SPF chickens, signals were detected in the cecum as early as 5 h, and on days 4 and 5, the cecum gave the strongest signal confirming it as the major site of *S. enteritidis* colonization. Interestingly, for the first time, they were able to visualize yolk sac infection occurred at day 4 post-infection. Mutants of the putative virulence determinants, *SPI-1, fur*, and *tonB*, showed a clear reduction in yolk sac infection. The lumazine protein (LumP) helped to improve the limit of detection. The availability of bioluminescent bacteria allows a novel approach to follow the colonization of pathogenic bacteria in chickens.

In summary, this Research Topic highlighted the latest research in combating bacterial diseases in poultry. It is vital to keep searching for effective treatments to reduce antibiotic use in farms to minimize the risk of developing and transferring resistance traits.

## Author contributions

All authors listed have made a substantial, direct, and intellectual contribution to the work and approved it for publication.
